# An overview of vaccine development for COVID-19

**DOI:** 10.4155/tde-2020-0129

**Published:** 2021-02-24

**Authors:** Seyed H Shahcheraghi, Jamshid Ayatollahi, Alaa AA Aljabali, Madhur D Shastri, Shakti D Shukla, Dinesh K Chellappan, Niraj K Jha, Krishnan Anand, Naresh K Katari, Meenu Mehta, Saurabh Satija, Harish Dureja, Vijay Mishra, Abdulmajeed G Almutary, Abdullah M Alnuqaydan, Nitin Charbe, Parteek Prasher, Gaurav Gupta, Kamal Dua, Marzieh Lotfi, Hamid A Bakshi, Murtaza M Tambuwala

**Affiliations:** ^1^Infectious Diseases Research Center, Shahid Sadoughi Hospital, Shahid Sadoughi University of Medical Sciences, Yazd, Iran; ^2^Department of Pharmaceutics & Pharmaceutical Technology, Yarmouk University, Irbid, Jordan; ^3^School of Pharmacy & Pharmacology, University of Tasmania, Hobart, Australia; ^4^Priority Research Centre for Healthy Lungs, School of Medicine & Public Health, The University of Newcastle, Callaghan, Australia; ^5^Department of Life Sciences, School of Pharmacy, International Medical University, Kuala Lumpur, Malaysia; ^6^Department of Biotechnology, School of Engineering & Technology, Sharda University, Greater Noida, Uttar Pradesh, India; ^7^Department of Chemical Pathology, School of Pathology, Faculty of Health Sciences & National Health Laboratory Service, University of the Free State, Bloemfontein, South Africa; ^8^Department of Chemistry, School of Science, GITAM Deemed to be University, Hyderabad 502329, India; ^9^Discipline of Pharmacy, Graduate School of Health, University of Technology Sydney, Ultimo, NSW 2007, Australia; ^10^Faculty of Pharmaceutical Sciences, Maharshi Dayanand University, Rohtak, India; ^11^School of Pharmaceutical Sciences, Lovely Professional University, Phagwara 144411, Punjab, India; ^12^Department of Medical Biotechnology, College of Applied Medical Sciences, Qassim University, Saudi Arabia; ^13^Departamento de Química Orgánica, Facultad de Química y de Farmacia, Pontificia Universidad Católica de Chile, Av. Libertador Bernardo O’Higgins, Santiago 340, Región Metropolitana, Chile; ^14^Department of Chemistry, University of Petroleum & Energy Studies, Dehradun 248007, India; ^15^School of Pharmaceutical Sciences, Suresh Gyan Vihar University, Jaipur, India; ^16^Discipline of Pharmacy, Graduate School of Health, University of Technology Sydney, Ultimo, NSW 2007, Australia; ^17^Department of Medical Genetics, School of Medicine, Shahid Sadoughi University of Medical Sciences, Yazd, Iran; ^18^Abortion Research Center, Reproductive Sciences Institute, Shahid Sadoughi University of Medical Sciences, Yazd, Iran; ^19^School of Pharmacy & Pharmaceutical Sciences, Ulster University, Coleraine, County Londonderry, Northern Ireland, BT52 1SA, UK

**Keywords:** COVID-19, COVID-19 vaccines, SARS-CoV-2

## Abstract

The COVID-19 pandemic continues to endanger world health and the economy. The causative SARS-CoV-2 coronavirus has a unique replication system. The end point of the COVID-19 pandemic is either herd immunity or widespread availability of an effective vaccine. Multiple candidate vaccines – peptide, virus-like particle, viral vectors (replicating and nonreplicating), nucleic acids (DNA or RNA), live attenuated virus, recombinant designed proteins and inactivated virus – are presently under various stages of expansion, and a small number of vaccine candidates have progressed into clinical phases. At the time of writing, three major pharmaceutical companies, namely Pfizer and Moderna, have their vaccines under mass production and administered to the public. This review aims to investigate the most critical vaccines developed for COVID-19 to date.

As demonstrated by COVID-19, new infections caused by RNA viruses which are subject to genetic changes and mutations will continue to create a significant global health danger [1–4]. Despite two past global coronavirus infection epidemics causing SARS and Middle East Respiratory Syndrome, the world remains unprepared to handle the current COVID-19 event effectively, as proven by the fact that COVID-19 has resulted in very many deaths in the world [5,6]. Although COVID-19 is a novel disease, MERS and SARS research has helped investigators understand how the human body reacts to coronaviruses and how the immune system’s response provides defence against the virus [7].

As with the process of drug development, vaccine progress for viral infections is tricky and typically is time-consuming. In the case of COVID-19 disease, the process is much more complicated due to the disease’s uncertain pathogenesis, the lack of availability of a validated animal model and the success of clinical trials on humans [8]. The right dosage and schedule for the vaccine can also be determined using limited human studies. After a single dose, some vaccines induce an adequate immune response, while others need a booster dose after a month or longer. This plan also prolongs the period of studies [9].

Over 56 verified effective candidate vaccines for COVID-19 are being produced in China, North America, Europe and Australasia. It is well recognized that vaccine manufacture is a lengthy and costly process [10,11]. Out of 78 established vigorous plans, 73 are now at preclinical phases [12].

This review discusses the most crucial candidate vaccines for the disease and reviews the latest studies in this field. We searched the official and preprint websites, the databases of Scopus, Medline, Web of Science, PubMed, Open Access Journals, LISTA (EBSCO) and Google Scholar to identify literature with the following terms: ‘COVID-19’ OR ‘2019-nCoV’ OR ‘SARS-CoV-2’ OR ‘Vaccine’.

## Vaccines for COVID-19

### Live weakened vaccine

In live vaccines, reverse genetic techniques have been effectively applied to inactivate the exonuclease effects of protein 14 (nsp14), a nonstructural peptide, and remove the envelope protein in the disease-causing virus [13]. The Bacille Calmette–Guérin (BCG) vaccine is a live vaccine that has been extensively utilized to prevent tuberculosis and leprosy since 1921 [14]. Scientists have concluded that the BCG vaccine will aid improvements in the immune system, thus decreasing SARS-CoV-2 infection rates. The efficacy of the BCG vaccine in minimizing the incidence of COVID-19 in different children’s hospitals in Western Australia is being tested in a variety of Phase III trials (NCT04327206). The effectiveness of BCG vaccination in increasing health among healthcare staff participating in COVID-19 patient care is being investigated by scientists from Radboud University of Netherlands (NCT04328441). These two studies are estimated to yield results by the end of 2022 [15]. Also, the avian infectious bronchitis virus (IBV) is a contagious chicken coronavirus, and the live IBV vaccine (strain H) has been suggested to be beneficial for SARS because the safety given by strain H is focused on blocking the production of antibodies and other immune responses. Therefore after evaluating its protection in monkeys, the avian IBV vaccine may be investigated as another choice for COVID-19 [16,17].

### DNA vaccine

An artificial DNA vaccine directing production of the coronavirus spike (S) protein, the main coronavirus surface antigen presently used in the clinical trial, has been developed to produce a synthetic DNA-based SARS-CoV-2 S protein candidate vaccine based on prior knowledge. The engineered design, INO-4800, leads to vigorous *in vitro* expression of the S protein. Efficient antibodies that neutralize SARS-CoV-2 infection inhibit S protein binding to the ACE2 receptor, and the circulation of SARS-CoV-2-targeted antibodies to the lungs was assessed following immunization of guinea pigs and mice with INO-4800. This study has verified that INO-4800 is a likely COVID-19 vaccine candidate [18,19].

### RNA vaccine

As shown in several preclinical investigations, the S protein is considered a key viral antigen for the progress of COVID-19 vaccines [19].

A new lipid nanoelement–mRNA-based vaccine, mRNA-1273, encodes the SARS-CoV-2 S protein, and started its first Phase I trial in the USA in March 2020. Successful technological progress in the production of vaccines has been demonstrated in the introduction of mRNA-based vaccines; however, the testing and development of vaccines as quickly and efficiently as possible to control COVID-19, which needs global co-operation, continues to present huge problems. A Phase I protection trial is being performed on one of the first vaccines, mRNA-1273. The newly invented encapsulated distribution of RNA and self-amplifying RNA method has helped to produce an mRNA effective vaccine with an improved success rate [20–23]. Scientists have also made accessible the security, immunogenicity and tolerability documents from a study (ClinicalTrials.gov identifier NCT04368728) carried out among individuals aged 18–55 years, who were selected to get two doses (10, 30 or 100 μg) of BNT162b1 during 3 weeks. BNT162b1 is a recombinant mRNA vaccine that encodes the S protein receptor-binding domain (RBD) of the SARS-CoV-2 virus. Nevertheless, for mRNA-based vaccines, some questions still need to be answered [24].

### Subunit vaccine

The antigen’s appearance in its most steady and efficient conformation has been one of the difficulties of using the protein subunit vaccine. Virus-like particles (VLPs) are comprised of several protein molecules capable of self-assembling into nanostructures that surround the capsid proteins inside themselves upon recombinant expression [25]. VLPs, which have a lipid membrane arising in the budding form from the cell membrane, may also be chimeric in origin, showing an envelope protein from another virus. In bacterial systems, mammalian cell lines and transgenic plants, a wide variety of advanced platforms express and correctly fold antigenic proteins. Moreover, because VLPs lack a genome of their own, they deliver a degree of stability comparable to that of nonreplicating vectors, thereby verifying the safety of the subunit vaccines and the effectiveness of the live vaccines [8].

Other subunit vaccines are based on MERS-CoV and SARS-CoV recombinant S and S1 proteins and have shown success in several studies [26–29]. Utilizing Tag technology, Clover Biopharmaceuticals is manufacturing a vaccine containing a viral S protein [30]. The SARS-CoV-2 RBD has been shown to have a considerably high binding affinity for the ACE2 receptor [31], signifying that RBD-based vaccines may be suitable for inhibition of COVID-19 infection. Several groups are currently developing RBD-based vaccines via global partnerships [32]. Nanoparticles related to pulmonary surfactant have been applied for generating immunity against influenza and can be utilized as adjuvant to increase the safety of SARS-CoV-2 subunit vaccines [33–35]. The helper T lymphocyte, B cell, adjuvant and cytotoxic T lymphocyte (CTL) epitopes are included in another vaccine construction. The evidence proposes that this vaccine is thermostable and nontoxic, and produces a stable cell immune response. Molecular and biological studies have confirmed the stability of the vaccine structure. This exceptional vaccine is produced by almost 30 extremely epitopes related to proteins that have notable importance in viral entrance, pathogenicity and host receptor identification [36]. Immunobioinformatics has also lately been utilized to classify important cytotoxic epitopes of CTL and B cells in the SARS-CoV-2 S protein. The relationships between these antigenic epitopes and MHC class I molecules related to them have been studied using molecular dynamics models and it has been discovered that the CTL epitopes connect via several contact places with grooves binding peptide of MHC class I, suggesting their ability to produce immune responses. These epitopes’ perfect features may make them suitable to become part of candidate COVID-19 vaccines [37]. The nucleocapsid (N) protein and the probable B cell epitopes of the MERS-CoV E protein have also been proposed as likely immunodefensive goals that stimulate immune responses [38,39].

Three vaccines were built using the designated epitopes in a study by docking method targeted to combat SARS-CoV-2. Three separate adjuvants (HABA, L7/12 protein and β-defensin) and various linkers (GPGPG, EAAAK and KK) were utilized at suitable locations to build these vaccines. The PADRE sequence is also a critical sequence applied in the production of vaccines; it can increase their efficacy, with high therapeutic effects and low toxicity. Furthermore, this sequence enhanced the CTLs response, therefore certifying effective immune responses. This approach can be used for the creation of a likely vaccine against COVID-19 [40,41].

### Vector-based vaccines

Viral vectors are commonly utilized together with virus vaccines, in which the genome of one virus is applied to transmit the antigen of another virus, facilitating the advancement of platform system for the creation of viruses. These tools are accessible to create vaccines on a wide scale. The disadvantages of these kinds of vaccines include a wide variety of purification methods, the need for accurate purity verification and virus action [42].

A COVID-19 vaccine using artificial antigen-presenting cells (aAPCs) was advanced by using genetically altered lentivirus, including the SARS-CoV-2 immune regulatory genes and minigenes, to modify the aAPCs as SARS-CoV-2 antigen-presenting. On 15 February 2020 a Phase I clinical trial of 100 applicants began, with an expected study finishing date of 31 December 2024 (NCT04299724). By altering dendritic cells with lentivirus vectors expressing SARS-CoV-2 minigene SMENP and immune regulatory genes, the lentiviral SMENP DC vaccine was designed and advanced. The Phase I trial of the vaccine, including almost 100 patients, began on 24 March 2020, and the assessed study end date will be 31 December 2024 (NCT04276896) [15,34,43,44].

A novel vaccine against COVID-19, ChAdOx1 nCOVID-19, is in the clinical phase; it was primarily advanced to inhibit MERS [12]. The base of the vaccine is an adenovirus vector and the S protein of SARS-CoV-2 [9]. It has been changed so that it cannot be produced in the body of humans. The genetic code for SARS-CoV-2 S protein synthesis has been inserted to enable the adenovirus to generate this protein after vaccination. The result is the creation of an antibody against the S protein [9].

The MERS-CoV S protein-expressing recombinant adenovirus vaccine stimulates bloodstream IgG, lung memory T cells and IgA when administered to BALB/c mice, and creates long-term immunity to the MERS virus, indicating that recombinant adenovirus vaccines can provide defense against the MERS-CoV virus [45]. Adenovirus type 5 (Ad5)-nCoV is the primary new genetically engineered vaccine for COVID-19 advanced by the Beijing Institute of Biotechnology and CanSino Biologics, China. As a vector, the vaccine uses replication-damaged Ad5 to express the SARS-CoV-2 S protein. This vaccine is presently in a successful Phase I clinical trial to examine its efficacy, immunogenicity and reactogenicity in a group of healthy individuals aged 18–60 years (NCT04313127). The study is planned to recruit 108 Wuhan participants who have tested negative for COVID-19 in the diagnostic experiments and is anticipated to be finished by 2022 [15].

Ad26-S forms the basis of another adenoviral vector-based COVID-19 vaccine [46]. As the seroprevalence of Ad26 has been shown to be almost 40% in humans, it is characteristically less immunogenic in comparison with Ad5 [47]; active immunity needs repeated heterologous or homologous vaccination, as has been revealed in Ad26-Ebola and Ad26-HIV vaccine studies in individuals [48,49]. However, an administration of a COVID-19 vaccine designed with an AD26 vector (Ad26.COV2.S) has presented strong safety in a nonhuman (primate) model of SARS-CoV-2 [50,51]. The rabies virus and the Gram-positive enhancer matrix – a bacterial vector – have also been utilized for expressing MERS-CoV S protein. Immune responses to these vaccines were tested for humoral and cellular immune reactions in BALB/c mice, which revealed that the rabies virus-based vaccine induces considerably higher cellular immunity rates and faster antibody responses than the Gram-positive enhancer matrix vector vaccines [52].

**Figure 1.**
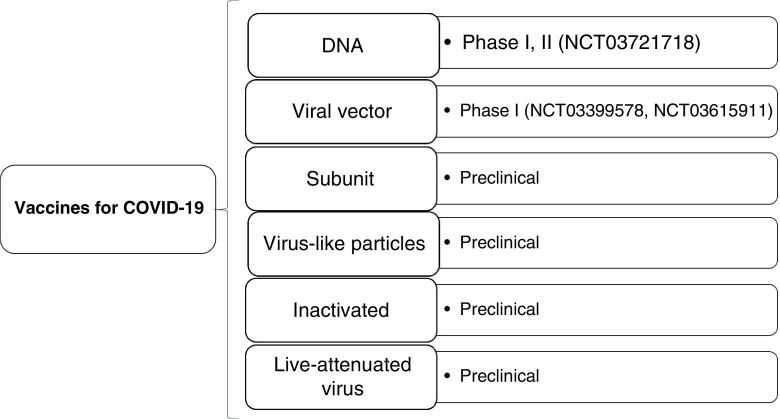
Clinical phases of COVID-19 vaccine candidates currently under investigation in human clinical trials.

Figure 1 shows the current trial phases of different vaccine types.

Vaccines go through Phase I trials to primarily evaluate their safety, determine the effective doses and investigate potential side effects in a limited number of volunteers. Phase II trials explore the vaccine candidate safety and efficacy in a larger group of volunteers. In Phase III, with very limited vaccine candidates, thousands of volunteers are used to evaluate the vaccine’s efficacy further and assess side effects on different population representatives. This also allows assessment of the various biotechnological methods that have been used to develop clinically viable COVID-19 vaccine candidates in the current pandemic, including DNA-based candidates, viral vectors, subunit vaccines, virus-like particles, inactivated and live-attenuated viruses [53].

### Inactivated SARS-CoV-2 vaccine

**Figure 2.**
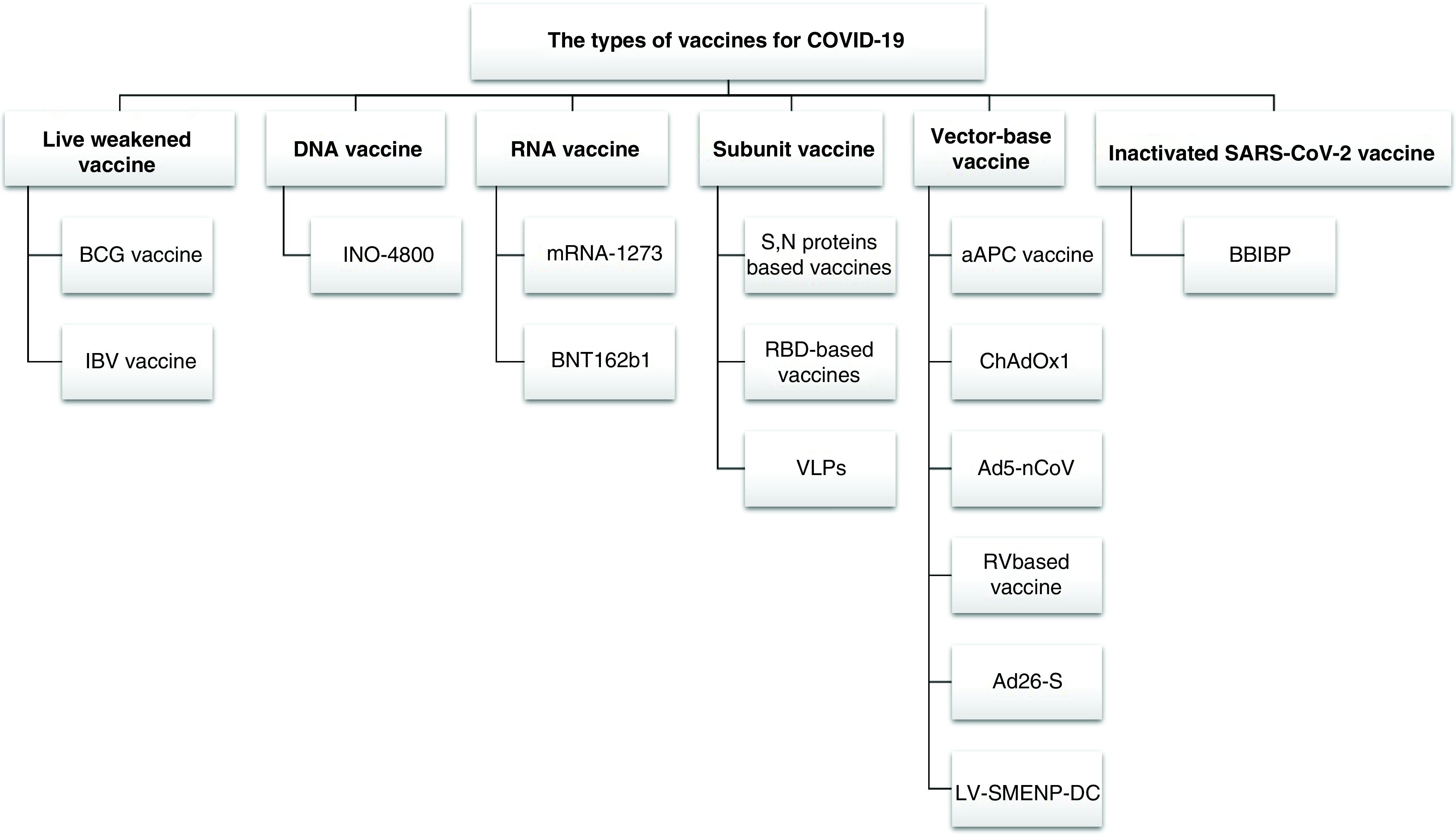
Summary of strategies for COVID-19 vaccine development compared with classical approaches.

Investigations have also demonstrated the ability of an inactivated vaccine candidate against COVID-19 (BBIBP-CorV) that stimulates the production of antibodies in rats, mice, rabbits and primates (rhesus macaques and cynomolgus monkeys) to protect against SARS-CoV-2. Two doses of BBIBP-CorV has given a highly effective defense against SARS-CoV-2 infection in rhesus macaques, without observable antibody-dependent infection. Furthermore, BBIBP-CorV shows efficient efficiency and good genetic stability [54]. Figure 2 shows the most important vaccine types being developed against COVID-19.

The first category of vaccine development includes approaches using the whole organism but inactivated through chemical or physical methods. BBIBP is an example of a vaccine using inactivated viral particles; such an approach has higher stability but requires stabilization of the viral structure, which complicates the vaccine manufacturing process. On the other hand, BCG and IBV were generated through the propagation of live attenuated viruses by cultivating particles under suboptimal growth conditions. INO-4800 represents a new and innovative direction of DNA-based vaccine production through recombinant DNA technology by encoding and expressing the target sequence in a suitable cell line via a viral-based vector. RNA is a relatively new technology for vaccine development; in essence, it utilizes the host body to translate the mRNA fragment of the spike protein to induce an immune response. The two candidates that have been developed so far by this method are mRNA-1273 and BNT162b1. Subunit vaccines contain only the antigenic epitopes (surface fragments) produced through recombinant DNA technologies, such as the RBD-based vaccine and the use of VLPs that elicit the immune response without the risk of developing the infection. The traditional approach for vaccine development is using viral vectors, in which a viral genome such as that of adeno-associated virus is used to deliver the antigen of another virus; some examples of this approach include aAPC vaccine, ChAdOx1, Ad5-nCoV, Ad26-S and LV-SMENP-DC.

### COVID-19 vaccines in action

The development of a safe vaccine takes years of research before the product can be deployed to clinical use. Typically after development, testing involves various stages [55]. Phase I, the preclinical phase, involves testing on cells and animals, then testing on a small number of people to confirm immune system stimulation [55]. Phase II testing involves testing in hundreds of people, including children and the elderly, further confirming safety in a different group of people [56]. Finally, the Phase III trial involves testing in thousands of people. In this phase, scientists give vaccines to the volunteers and wait to see how many become infected in the placebo and vaccine group. These trials typically determine whether the vaccine protects against the virus, with or without side effects. Phase III trials are large enough to reveal the efficacy rate and rare side effects. Based on Phase III, regulatory authorities decide to authorize the vaccine initially for the limited approval [57]. Regulators review the complete trial results and plans for manufacturing the vaccine and decide whether to give it full approval.

These testing steps usually take years to complete, and developers must adhere to the necessary protocols to develop safe and effective vaccines. However, in the present pandemic scientists worldwide are in a race to produce safe and effective vaccines in record time, without taking shortcuts on the protocol. Around 70 vaccines developed by universities and companies are in clinical trials, and around 20 of them have reached the final testing stage [58]. At the time of writing, two vaccines, developed by Moderna and Pfizer, have received full approval in different countries for mass immunization [59].

Both the Moderna and Pfizer vaccines are mRNA-based vaccines that tell cells to make the SARS-CoV-2 S protein, to generate an immune response. The Moderna mRNA-1273 vaccine was jointly developed by the Massachusetts-based biotechnology company Moderna, Inc. and the US National Institute of Allergy and Infectious Diseases. This vaccine is a formulation of nanoparticles in which mRNA encoding the full-length spike protein of the coronavirus is encapsulated inside liposomes. The Phase III trial of this vaccine, also known as the COVE trial, consisted of randomized, observer-blinded, placebo-controlled trials conducted at 99 centers across the USA [60]. Volunteers at high risk for SARS-CoV-2 infection were randomly assigned in a 1:1 ratio to receive two intramuscular injections of mRNA-1273 (100 μg) or placebo with a gap of 28 days. The trial’s primary end point was prevention of COVID-19 illness with onset at least 14 days after the second injection in participants who had not previously been infected with SARS-CoV-2 [60].

More than 96% of participants received both the doses, while 2.2% of volunteers were previously infected with SARS-CoV-2, as confirmed by serologic testing. Eleven participants in the mRNA-1273 group and 185 in the placebo group were confirmed as having symptomatic COVID-19 illness. Severe symptoms of COVID-19 were observed in 30 participants, and there was one fatality, all from the placebo group. Serious adverse events were rare in the mRNA-1273 group, and the overall efficiency of the vaccine was observed to be 94.1% [60].

Another vaccine (BNT162b2) for which the US FDA gave emergency use authorization for the prevention of COVID-19 was jointly developed by the major pharmaceutical company Pfizer and its German partner BioNTech. This vaccine was authorized to be used in individuals 16 years of age and older. This vaccine has been approved in Britain, the USA, the EU and Canada [61].

In the multinational, placebo-controlled, observer-blinded, pivotal efficacy Phase III trial of BNT162b2, persons aged 16 years or older were randomly assigned in a 1:1 ratio to receive two doses (30 μg per dose) at 21 days apart [61]. The two groups into which the volunteers were assigned were BNT162b2 and the placebo group. Similar to the Moderna vaccine, BNT162b2 is also formulated as nanoparticles composed of different lipids, encapsulating mRNA that codes for the membrane-anchored SARS-CoV-2 full-length spike protein against which the immune cells initiate the immune response [61]. The primary end points of the trials were the efficacy of the vaccine against laboratory-confirmed COVID-19 and safety. Out of 43,548 volunteers, 21,720 received BNT162b2 and 21,728 received placebos. A total of eight cases of COVID-19 in the BNT162b2 group and 162 cases in the placebo group were observed, confirming overall 95% effectiveness in preventing COVID-19. Similar efficiency of the vaccine was observed in different subgroups defined by sex, race, ethnicity, baseline BMI, age and the presence of coexisting conditions. Among 10 confirmed cases of COVID-19, nine were from the placebo group and one was from the vaccinated group. The incidence of serious adverse events was rare and was identical in both groups [61].

In a head-to-head comparison of both vaccines, each vaccine is effective after both doses. The Pfizer and Moderna vaccines were found to be 95 and 94.1% effective, respectively, after the second dose in adults aged 16 years and older. The two vaccines’ potential side effects are almost identical and include injection site pain, swelling, redness, tiredness, headache, muscle pain, joint pain, chills, fever and nausea/vomiting. However, the vaccines are a bit different when it comes to the question of stability and transportation. The Pfizer vaccine is only stable below −70°C and hence needs to be shipped or transported in temperature-controlled thermal shippers that keep the temperature at this level. The vaccine can be stored in this condition for up to 10 days. For storage of more than 10 days, ultra-low temperature is required.

On the other hand, the Moderna vaccine requires a temperature of -20°C for shipping and can be stored at 2–8°C for 30 days. It is stable for 6 months if stored at -20°C and for up to 12 h at room temperature. The significant difference in the two vaccines’ stability is the result of the different lipids used for their nanoparticle formulations.

## Discussion & conclusion

A novel β-coronavirus, SARS-CoV-2, appeared in Wuhan (Hubei Province, China) in December 2019, where it was identified as responsible for the COVID-19 infection [62]. To end the global SARS-CoV-2 pandemic, a vaccine is needed. Any vaccine that is reliable, effective, permanent and accessible to a large population is a good candidate. However, viral particles can mutate, which can make vaccines ineffective; therefore it is important to create a secure and reliable vaccine in advance for future outbreaks of SARS-CoV-2 variants. Most vaccine approaches tend to produce antibodies that neutralize specific proteins such as the S protein. SARS-CoV-2-neutralizing vaccines dependent on RBD S protein will efficiently be created. Apart from antigens, certain supplementary vaccine components can increase the immune response and decrease the amount of antigen necessary for each vaccine dose. The design of a vaccine offering active protective immunity will be the most successful long-term strategy for preventing potential outbreaks of this virus. As of April 2020, 115 vaccine candidates were included in the international COVID-19 vaccine research and design landscape [63]. The most crucial candidate vaccines include viral vector vaccines, nucleic acid-based vaccines, aAPC vaccines, viral proteins and live vaccines [12,20,64,65]. Among all the vaccines in the clinical trial process as options for SARS-CoV-2, the RNA-based vaccines appear to be more effective than other vaccines since large amounts of vaccine are amenable to low-budget development. Though harmless in clinical trials, the immune response caused by an antigen whose production is stimulated by an RNA-based vaccine is lesser than that detected in animal models [66,67]. Like RNA-based vaccines, DNA vaccines are easy to create, inexpensive, and offer better safety and efficacy and lengthy immunogenic persistence. Even so, because they failed to elicit a sufficiently robust immune response to be safe, they have not been licensed for human usage. Instead, vaccines based on extremely immunogenic vectors have been revealed to produce effective immune responses.

Nevertheless, the application of an adenovirus vector to transfer the gene encoding the goal protein, as in the case of Ad5 with the expression of S protein, increases some anxieties associated with immunity against adenovirus types in humans [68]. Finally, lentiviral vectors derived from viral proteins have been associated with some protection issues related to the possible risk of mutagenesis [69]. A thorough study of the immunological associations with SARS-CoV-2 involves the production of an active vaccine; however, most approaches would not serve the required urgency because of the disease epidemic’s severity.

Consequently, a numerical forecast helps direct scientists to produce a vaccine and help monitor the disease. Creating a vaccine is a lengthy and costly process with high rejection rates, and the manufacture of a safe commercial vaccine usually takes many candidates and years [36]. Finally, testing and making COVID-19 vaccines is expected to increase the ‘inevitability of realization’ of indicating vaccine effectiveness and monitoring COVID-19 infection, death and the pandemic itself [70].

## Future perspective

The progress of a vaccine signifies the perfect therapeutic option for the COVID-19 epidemic, but despite the development of three vaccines, it can still be a long route to normality.

The best candidate vaccines include vector-based vaccines, nucleic acid vaccines, live vaccines, viral proteins and aAPC vaccines. Currently, RNA-based and vector-based vaccines appear to be more favorable than other types. However, we still need to watch this space for future developments. There is a need to develop safe and effective therapeutics for respiratory inflammation-linked viral infection; this strategy could avoid future events similar to the current pandemic caused by COVID-19.

**Executive summary**

- New innovative technologies have been developed for vaccines for the first time in response to the COVID-19 pandemic.
- Emergency use authorization has been granted to four leading vaccine candidates to be used globally.
- Currently, under development, there are over 200 COVID-19 vaccine candidates in various stages of trials.
- Vaccines traditionally take a long time to develop; the development of multiply efficient and highly effective vaccines against COVID-19 is a remarkable scientific achievement.
- Dozens of vaccine candidates fit into eight categories, with four currently being given to individuals globally.
- Hopes are rising that we will soon see the end of the pandemic with the roll-out of COVID-19 vaccines.
